# Secondary Spontaneous Pneumothorax in a Patient With Interstitial Lung Disease Undergoing Routine Outpatient Pulmonary Function Tests

**DOI:** 10.7759/cureus.55675

**Published:** 2024-03-06

**Authors:** Kristina Akopyan, Raaed Zafar, Ibrahim Faruqi

**Affiliations:** 1 Internal Medicine, University of Florida, Gainesville, USA; 2 Medicine, University of Texas at Dallas, Dallas, USA; 3 Pulmonary and Critical Care Medicine, University of Florida, Gainesville, USA

**Keywords:** pleurodesis, chest tube, pulmonary function test, interstitial lung disease, pneumothorax (ptx)

## Abstract

We present the case of a 64-year-old female with a past medical history significant for unclassified interstitial lung disease (ILD) from suspected hypersensitivity pneumonitis secondary to chronic mold exposure with steroid responsiveness and prior pneumothorax. The patient developed shortness of breath and pleuritic chest pain after undergoing routine outpatient pulmonary function tests (PFTs). She was immediately transferred to the emergency department and found to have a moderate left basilar pneumothorax. She underwent emergent surgical chest tube placement followed by doxycycline pleurodesis. Repeat chest imaging showed inadvertent retraction of the chest tube and extensive subcutaneous emphysema. The surgical chest tube was replaced by a pigtail catheter with an improvement of subcutaneous emphysema. This case demonstrates the development of a rare but serious complication of pneumothorax that could occur in patients who have ILD undergoing routine PFTs. Clinicians should be aware of this risk when patients who have ILD present for PFTs and counsel them to seek immediate medical attention if they develop signs of acute onset dyspnea after performing PFTs.

## Introduction

A pneumothorax is defined as an accumulation of air in the pleural space. A pneumothorax could be traumatic or spontaneous. Spontaneous is further divided into primary (absence of an underlying lung disease) and secondary (presence of an underlying lung disease) [1]. Secondary spontaneous pneumothorax (SSP) in ILD is a life-threatening complication that can lead to hemodynamic collapse. Patients with concomitant pneumomediastinum have a worse prognosis than those without [2]. PFT is a tool that is used to evaluate, monitor, and manage patients with lung disease that is suspected or established. This tool is used to evaluate airflow, lung volumes, and diffusion capacity. Obstructive lung disease is defined by decreased flow relative to lung volume, which is reflected by the forced expiratory volume in the first second (FEV1) to the forced vital capacity (FVC) ratio. Restrictive lung disease is defined by reduced vital capacity (VC) with normal flow rates [3]. Here, we present a case of SSP in a patient with ILD undergoing routine outpatient PFTs.

## Case presentation

A 64-year-old female with a past medical history significant for unclassified ILD from suspected hypersensitivity pneumonitis secondary to chronic mold exposure with steroid responsiveness, spontaneous pneumothorax unsuccessfully treated with talc pleurodesis, followed by mechanical pleurodesis, and subsequent pneumothorax after cryobiopsy of the lung treated by an unsuccesful blood patch pleurodesis, followed by successful temporary endo bronchial valve placement, was undergoing routine outpatient PFTs. Upon developing acute onset of shortness of breath and pleuritic chest pain, she was immediately transferred to the emergency department. 

On physical examination, she was saturating 94% on a non-rebreather mask at 15 L/min, blood pressure was 143/105 mm Hg, respirations were 20 breaths per minute, pulse was 86 beats per minute, and temperature was 98.1 degrees Fahrenheit. She was found to be in mild distress. She had a normal pulse with regular rate and rhythm and no murmurs on auscultation. She had diminished breath sounds on the left with no wheezing or crackles noted. Her abdomen was non-distended and non-tender, and there was no guarding or rigidity. The skin was warm and dry. She was alert and oriented with no focal deficits observed. On blood tests, the complete blood count (CBC) and basic metabolic panel (BMP) were unremarkable.

**Figure 1:**
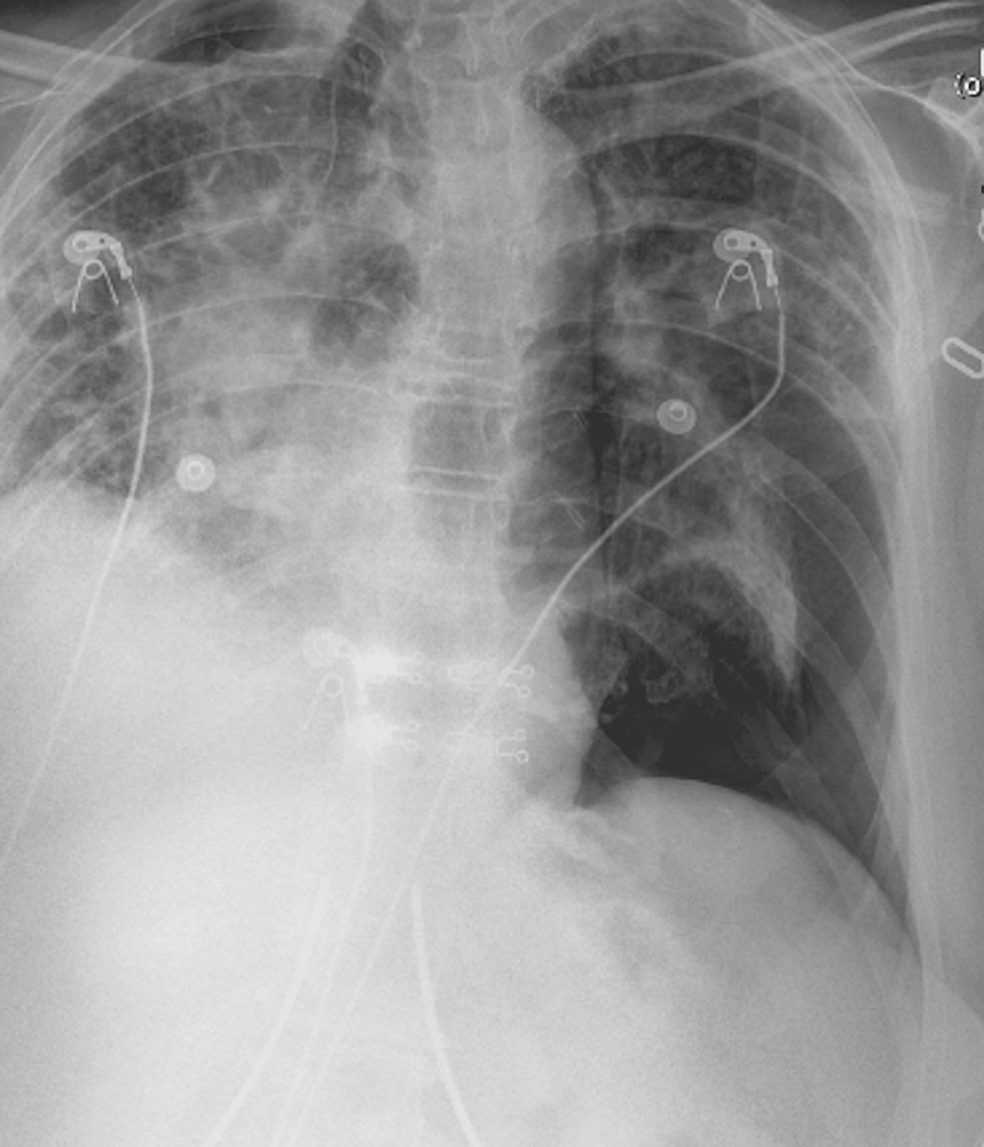
Chest X-ray (CXR) showing a moderate left basilar pneumothorax

Chest X-ray (CXR) revealed a moderate left basilar pneumothorax (Figure 1).

**Figure 2:**
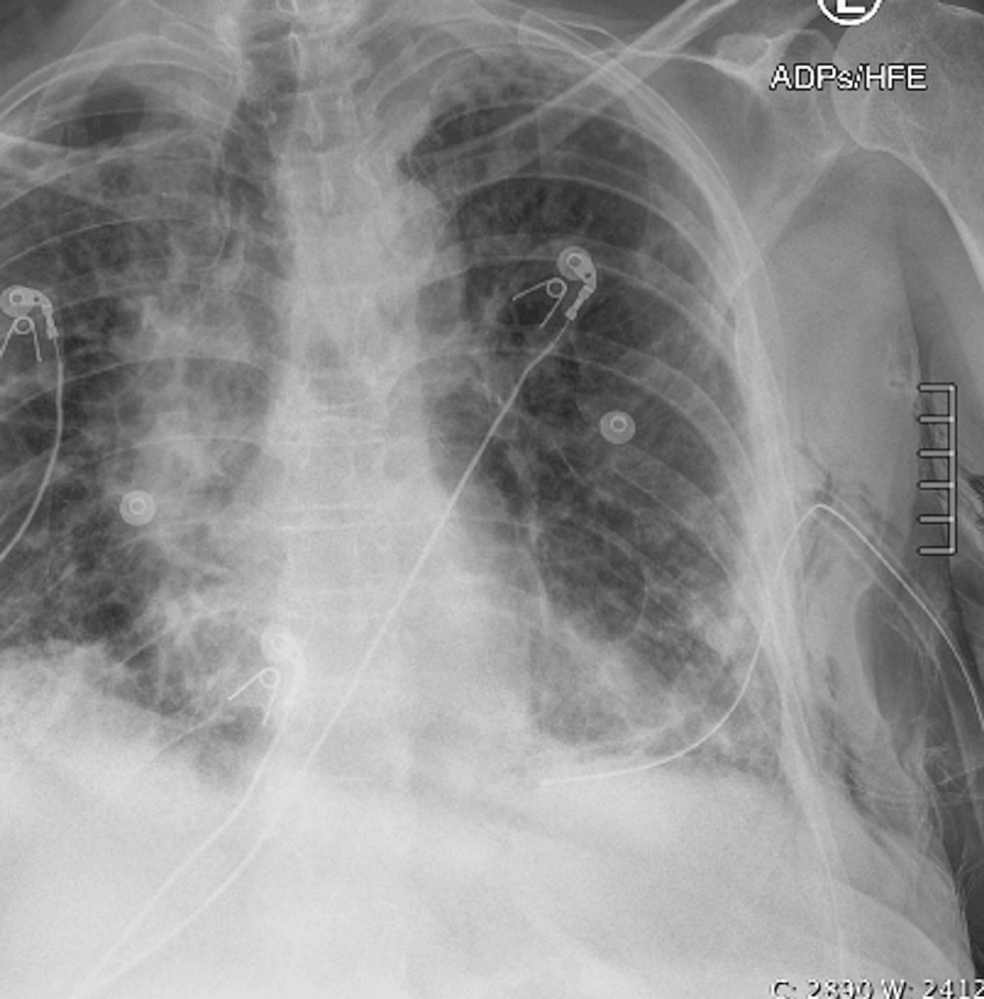
Chest X-ray (CXR) showing placement of the left thoracostomy tube with near complete resolution of the left basilar pneumothorax. There is a small amount of subcutaneous emphysema at the left lateral chest wall.

A 28 French surgical chest tube was placed with the wall suction placed at -20 cm H2O. Repeat CXR demonstrated near complete resolution of the left basilar pneumothorax (Figure 2).

**Figure 3:**
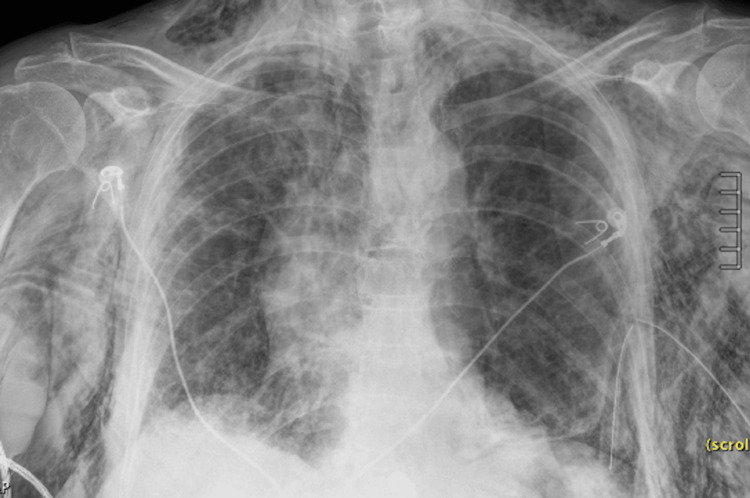
CXR showing interval development of extensive subcutaneous emphysema and pneumomediastinum. There is also migration of the left chest tube with a sentinel hole near the left chest wall and a new left basilar pneumothorax.

Doxycycline pleurodesis was done with follow-up chest X-ray demonstrating inadvertent retraction of the chest tube with a sentinel port along the chest wall and extensive subcutaneous emphysema (Figure 3).

**Figure 4:**
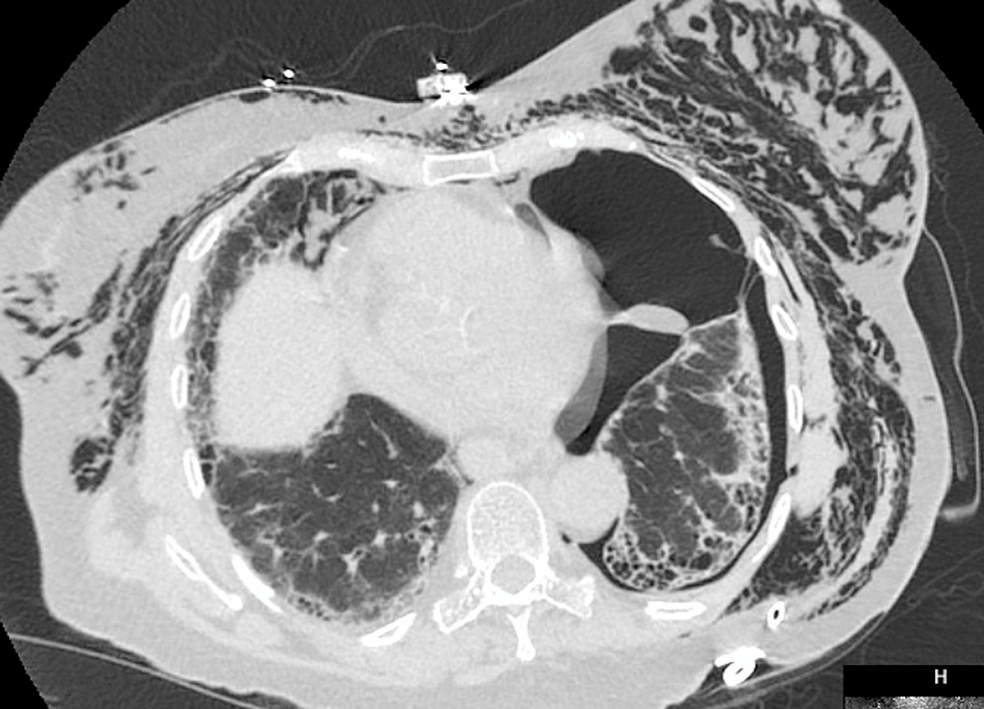
CT chest showing moderate left basilar pneumothorax with a rightward shift of the mediastinum. The thoracostomy tube appears appropriately positioned. There is extensive pneumomediastinum and chest wall subcutaneous emphysema extending into bilateral neck and shoulder fascial planes.

The surgical chest tube was removed, and a pigtail catheter was placed on the left side. CT chest revealed that despite a well-positioned left thoracostomy tube, there was a persistent left pneumothorax and pockets of air surrounded by fibrotic pleural tissue (Figure 4).

**Figure 5:**
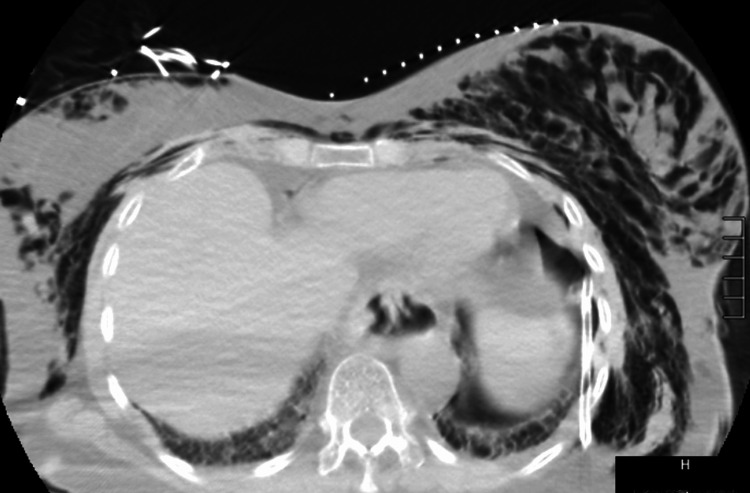
CT chest showing near complete resolution of the left basilar pneumothorax with the pigtail catheter in place

Because of persistent pneumothorax, wall suction was increased to -30 cm H2O, and a second chest tube was attempted by interventional radiology, but there was no good pocket for placement. The patient was only intermittently requiring oxygen, and she had improvement in left-sided pneumothorax and subcutaneous emphysema demonstrated by repeat CT chest (Figure 5).

The patient requested a transfer to an outside hospital to continue treatment near family, and she was then transferred with a left chest tube in place.

## Discussion

There are lung-related abnormalities that have been found to predispose people to having a pneumothorax. These include subpleural blebs and bullae, emphysema-like changes, chronic small airway inflammation, and visceral pleural porosity [4]. The most common underlying disorders associated with SSP are chronic obstructive pulmonary disease (COPD), cystic fibrosis, tuberculosis, lung cancer, and HIV-associated pneumocystis carinii pneumonia. Pneumothorax can also be associated with ILD, such as idiopathic pulmonary fibrosis (IPF), sarcoidosis, lymphangioleiomyomatosis, and connective tissue diseases, such as rheumatoid arthritis, Marfan syndrome, and Ehlers-Danlos syndrome. The incidence of SSP is similar to that of primary spontaneous pneumothorax (PSP). The incidence is reported as 7.4 to 18 cases per 100,000 each year in males and 1.2 to six cases per 100,000 each year in females [5]. The frequency of SSP in patients with ILD is reported as 12.9-20.2% [6]. Our patient with a past medical history of ILD had a major risk factor of developing pneumothorax in the past, even though she was only undergoing routine PFTs.

The pathophysiology of a pneumothorax can be explained by a change in the pressure gradient inside the thorax. The pressure of the pleural space is negative compared to the pressure of the atmosphere. The lung expands outward as the chest wall expands because there is surface tension between the parietal and visceral pleurae. The lung will collapse due to elastic recoil. Air will fill the space between the alveoli and pleural space when there is communication between them. This will change the gradient causing the pneumothorax to enlarge and the lung to become smaller due to this vital capacity [7]. Patients with ILD are known to have reduced lung volumes, poor gas exchange, and impaired cardiovascular function, which leads to exercise limitation [8]. ILD will result in characteristic changes on PFTs, which include reductions in the total lung capacity (TLC), forced vital capacity (FVC), and diffusion capacity for carbon monoxide (DLCO) [9].

The clinical presentation of pneumothorax can vary from being asymptomatic to having chest pain and shortness of breath. A tension pneumothorax could lead to obstructive shock with severe hypotension and even death. On physical examination, patients who have pneumothorax may have tachycardia, tachypnea, and hypoxia. There could also be hyper-resonant percussion noted and diminished or absent breath sounds. Tension pneumothorax would have findings of cyanosis, jugular venous distention, respiratory failure, and cardiac arrest [7]. Our patient immediately had chest pain and shortness of breath following her routine PFTs. On physical examination, she was found to have tachypnea and diminished breath sounds. It was crucial to consider pneumothorax in the differential diagnosis given her history of ILD and prior pneumothorax with her presenting symptoms.

Ultrasound can be used to diagnose a pneumothorax. For example, the presence of lung sliding indicates the visceral pleura is adhering to the parietal pleura. If air is present between these pleura, then there is the absence of lung sliding, which is indicative of a pneumothorax [10]. A pneumothorax can also be identified on a radiograph as increased lucency at the upper quadrant of the abdomen, sharp definition of the anterior diaphragmatic surface, or a wide and deep costophrenic angle, the deep sulcus sign [11]. A CT scan of the chest can also be used and is especially useful in the diagnosis of malpositioned chest tubes, which can be difficult to assess by radiography [12].

The treatment that is most frequently performed for patients with SSP and ILD is pleurodesis via a chest tube. Talc is considered the most effective and widely used sclerosing agent. However, this could also cause an acute exacerbation of ILD or acute respiratory distress syndrome and should be used with caution. A retrospective study of 180 patients with SSP caused by ILD evaluated the efficacy of pleurodesis and found that 64.2% were cured, 24.5% died during the clinical course, and 11.3% developed a recurrent pneumothorax following discharge [6]. Our patient had placement of a surgical chest tube, followed by doxycycline pleurodesis and subsequent development of subcutaneous emphysema, and required placement of a pigtail catheter. However, her pneumothorax and subcutaneous emphysema did improve after the wall suction was increased. The same retrospective study found that surgery was associated with very good outcomes with 24 out of 25 patients who received surgery being cured with no recurrence of pneumothorax [6]. This may reflect the future discussions of surgical indications for these patients who have SSP and ILD.

## Conclusions

This case describes a woman with a past medical history of ILD who developed pneumothorax after undergoing routine outpatient PFTs. Her clinical course is complicated by pneumomediastinum and worsening pneumothorax requiring the removal of a surgical chest tube and placement of a pigtail catheter. The case emphasizes the importance of being cognizant of the potential for developing a pneumothorax even in patients with ILD undergoing routine PFTs, especially in those with underlying risk factors. It is vital to have a low threshold for suspicion of pneumothorax when patients with ILD present for routine PFTs and develop symptoms of chest pain or shortness of breath. Clinical presentation may vary from being asymptomatic to having severe symptoms of hypotension and respiratory failure in the case of tension pneumothorax. Our patient immediately received clinical attention and had placement of a chest tube with pleurodesis with eventual improvement in her pneumothorax. It is imperative for clinicians to keep in mind that even with routine testing of lung function, severe complications may arise in this patient population.
